# Alterations in the oral microbiome of individuals with a healthy oral environment following COVID-19 vaccination

**DOI:** 10.1186/s12903-022-02093-6

**Published:** 2022-03-03

**Authors:** Osamu Uehara, Yoshihiro Abiko, Toshiyuki Nagasawa, Tetsuro Morikawa, Daichi Hiraki, Fumiya Harada, Yutaka Kawano, Seiko Toraya, Hirofumi Matsuoka, Durga Paudel, Shintaro Shimizu, Koki Yoshida, Masahiro Asaka, Yasushi Furuichi, Hiroko Miura

**Affiliations:** 1 Division of Disease Control and Molecular Epidemiology, Department of Oral Growth and Development, School of Dentistry, Health Sciences University of Hokkaido, 1757 Kanazawa, Ishikari-Tobetsu, Hokkaido 061-0293 Japan; 2 Division of Oral Medicine and Pathology, Department of Human Biology and Pathophysiology, School of Dentistry, Health Sciences University of Hokkaido, 1757 Kanazawa, Ishikari-Tobetsu, Hokkaido 061-0293 Japan; 3 Division of Advanced Clinical Education, Department of Integrated Dental Education, School of Dentistry, Health Sciences University of Hokkaido, 1757 Kanazawa, Ishikari-Tobetsu, Hokkaido 061-0293 Japan; 4 Division of Reconstructive Surgery for Oral and Maxillofacial Region, Department of Human Biology and Pathophysiology, School of Dentistry, Health Sciences University of Hokkaido, 1757 Kanazawa, Ishikari-Tobetsu, Hokkaido 061-0293 Japan; 5 Division of Oral and Maxillofacial Surgery, Department of Human Biology and Pathophysiology, School of Dentistry, Health Sciences University of Hokkaido, 1757 Kanazawa, Ishikari-Tobetsu, Hokkaido 061-0293 Japan; 6 Institute of Preventive Medical Science, Health Sciences University of Hokkaido, Ainosato 2-5, Kita-ku, Sapporo, Hokkaido 002-8072 Japan; 7 Advanced Research Promotion Center, Health Sciences University of Hokkaido, 1757 Kanazawa, Ishikari-Tobetsu, Hokkaido 061-0293 Japan; 8 Division of Periodontology and Endodontology, Department of Oral Rehabilitation, School of Dentistry, Health Sciences University of Hokkaido, 1757 Kanazawa, Ishikari-Tobetsu, Hokkaido 061-0293 Japan

**Keywords:** COVID-19, Vaccine, 16S rRNA, Oral microbiome, Healthy oral environment

## Abstract

**Background:**

Several reports suggest that the microbiome of the digestive system affects vaccine efficacy and that the severity of coronavirus disease (COVID-19) is associated with decreased diversity of the oral and/or intestinal microbiome. The present study examined the effects of a severe acute respiratory syndrome coronavirus 2 (SARS-CoV-2) mRNA vaccine on the oral microbiome.

**Methods:**

Forty healthy Japanese oral healthcare personnel were recruited, and unstimulated saliva was collected before vaccination, after the 1st vaccination, and after the 2nd vaccination. Genomic DNA was extracted from saliva samples, and PCR amplicons of the 16S rRNA gene were analyzed using next-generation sequencing. Microbial diversity and composition were analyzed using Quantitative Insights into Microbial Ecology 2. In addition, alterations in microbial function were assessed using PICRUSt2.

**Results:**

SARS-CoV-2 mRNA vaccination significantly increased oral bacterial diversity and significantly decreased the proportion of the genus *Bacteroides*.

**Conclusions:**

The SARS-CoV-2 mRNA vaccine alters the oral microbiome; accordingly, vaccination might have beneficial effects on oral health.

## Background

The oral cavity is one of the most crucial routes of severe acute respiratory syndrome coronavirus 2 (SARS-CoV-2) infection, and the virus is present in high concentrations in the saliva of patients with coronavirus disease 2019 (COVID-19) [1]. Several reports have suggested that COVID-19 patients exhibit dysbiosis of the oral microbiome [2–4]. Furthermore, a decrease in the alpha diversity of the oral microbiome is correlated with the severity of COVID-19, and oral dysbiosis is associated with increased levels of inflammatory cytokines and a decreased IgA response [5]. However, these cross-sectional studies did not explain whether dysbiosis of the oral microbiome was the cause of or resulted from COVID-19.

Vaccination is a crucial strategy in the fight against COVID-19, and its application is increasing worldwide. Though there is a possibility of alterations in the oral and intestinal microbiomes of vaccinated individuals, these microbiomes have not yet been analyzed [6]. Alterations in the microbiome might provide some beneficial information for understanding the health conditions of vaccinated individuals. However, it is also speculated that changes in the oral microbiome might directly affect oral health conditions, in addition to possibly modifying susceptibility to COVID-19 [2–4]. Therefore, potential environmental factors that might affect the oral microbiome composition should be excluded as much as possible to investigate the oral microbiome. In this study, we collected saliva from participants working as dental professionals after confirming their oral health conditions and systemic health and observed alterations in their oral microbiome following COVID-19 vaccination.

## Methods

### Data collection

Ethical approval for this study was granted by the Ethics Committee of the Institute of Preventive Medical Science, Health Sciences University of Hokkaido (Approval no. 2021–001). The methods used in the experiments were conducted in accordance with the Declaration of Helsinki. We enrolled 40 faculty and staff members of the Health Sciences University of Hokkaido who were scheduled to receive COMIRNATY® (Pfizer, NY, USA; BioNTech SE, Mainz, Germany). All individuals agreed to participate in this study and provided written informed consent. Individuals who were undergoing dental treatment or who had systemic conditions or smoking habits were excluded from the study. None of the individuals had contracted COVID-19. Among the participants, there were 22 males (mean ± SD years; 38.4 ± 12.3) and 18 females (32.2 ± 7.7). The number of participants in each age group was as follows: 20–29 years (n = 15); 30–39 years (n = 16); and over 40 years (n = 9). The decayed, missing, and filled teeth (DMFT) index was used to assess tooth condition according to the WHO-recommended criteria. According to the DMFT index, carious teeth received the letter D, missing teeth received the letter M, and filled or processed teeth that had not rotted received the letter F. DMFT was examined for 28 teeth, excluding the third molars. Periodontal examinations were performed using the WHO criteria [7]. The periodontal status of participants was recorded using the Community Periodontal Index (CPI). The index teeth (11, 16, 17, 26, 27, 31, 36, 37, 46, and 47) were examined. The scores adopted for CPI evaluation were as follows: 0, healthy periodontium; 1, presence of bleeding on probing; 2, presence of calculus; 3, 4–5 mm pocket; 4, ≥ 6 mm pocket.

### Sample collection

**Fig. 1:**
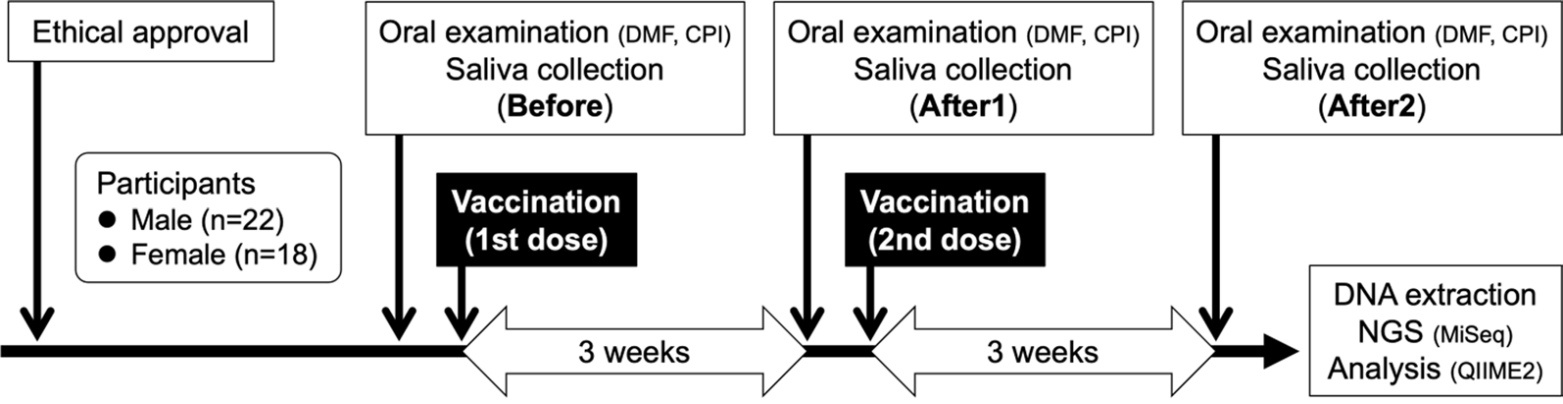
Schedule of the oral examination and saliva collection. Forty faculty and staff who were scheduled to receive COMIRNATY® were recruited. Saliva samples were collected before vaccination (Before), 3 weeks after the first inoculation (After1), and 3 weeks after the second inoculation (After2). The samples were collected from the participants at their homes when they woke up; the participants were instructed to avoid gargling, eating, drinking, and brushing their teeth before sample collection

Saliva samples were collected in conical tubes before vaccination (Before), 3 weeks after the first inoculation (After1), and 3 weeks after the second inoculation (After2). The samples were collected from the participants at their homes after they woke up. The participants were instructed to avoid gargling, eating, drinking, and brushing their teeth before sample collection (Fig. 1).

### DNA extraction and sequencing

Genomic DNA was extracted from saliva samples using DNeasy Blood & Tissue Kits (Qiagen, Hilden, Germany), according to the manufacturer’s instructions. Amplicon PCR was performed to target the V3–V4 regions of the bacterial 16S ribosomal RNA (rRNA) gene [8, 9]. Sequencing libraries of the V3–V4 region were generated according to the 16S Metagenomic Sequencing Library Preparation instructions (Illumina, San Diego, CA, USA). In brief, KAPA HiFi HS ReadyMix (Nippon Genetics, Tokyo, Japan) and V3–V4 region primers (forward, 5′-TCGTCGGCAGCGTCAGATGTGTATAAGAGACAGCCTACGGGNGGCWGCAG-3′ and reverse, 5′-GTCTCGTGGGCTCGGAGATGTGTATAAGAGACAGGACTACHVGGGTATCTAATCC-3′) were used for the amplicon PCR, and KAPA HiFi HS ReadyMix and Nextera XT index kits (Illumina) were used for the index PCR. The library was diluted, mixed with PhiX (Illumina), and then sequenced using an Illumina MiSeq system with a MiSeq reagent kit v3 (600 cycles, Illumina). The sequencing depth was determined to be 14,000 reads from alpha rarefaction.

### Analysis of sequencing data

Metagenomic sequencing data were analyzed using the software package Quantitative Insights into Microbial Ecology 2 (QIIME2 v2020.2) against the 16S rRNA gene sequences that were assigned to the 16S rDNA database (Greengenes v13.8) [8, 9]. Analysis of the amplicon sequence data employed the DADA2 pipeline. Alpha diversity was estimated using the observed OTUs, Faith’s phylogenetic diversity (PD), and Shannon index. Statistical significance was set at p < 0.05. Beta diversity was evaluated based on UniFrac distances representing the fraction of the branch length of the phylogenetic tree shared between the groups. Three-dimensional principal coordinate analysis (PCoA) was used to generate UniFrac scatterplots to visually compare microbial compositions across groups. The differences in bacterial communities among the Before, After1, and After2 groups were analyzed using the unweighted and weighted UniFrac distance metric. Permutational multivariate analysis of variance (PERMANOVA) was used with the unweighted and weighted UniFrac distance matrix to determine significant differences in microbial communities between the different groups. Statistical significance was set at p < 0.05. All results are presented as uncorrected p-values. Significant differences in microbial taxon abundance among the Before, After1, and After2 groups were analyzed using the analysis of composition of microbiomes (ANCOM) in QIIME2. The final significance was expressed as the empirical distribution of W. Feature volatility analysis was performed to determine the linear mixed-effects (LME) model results for bacterial taxonomy. Several bacterial taxa were selected for subsequent analysis, depending on their importance, which were analyzed using the LME model to detect important bacterial taxonomies [10]. The phylogenetic investigation of communities by reconstruction of unobserved states (PICRUSt2) software was used to predict functional abundances based only on marker gene sequences. The statistical analysis of metagenomic profiles (STAMP) software package was used to analyze the PICRUSt2 results. Welch's t-test was used for two-group comparisons, and the Welch's inverted confidence interval method was used to calculate the confidence interval. The Benjamini–Hochberg false discovery rate method was used to calculate the adjusted p-value (p < 0.05).

## Results

### Oral examination (DMFT index and CPI)

**Fig. 2:**
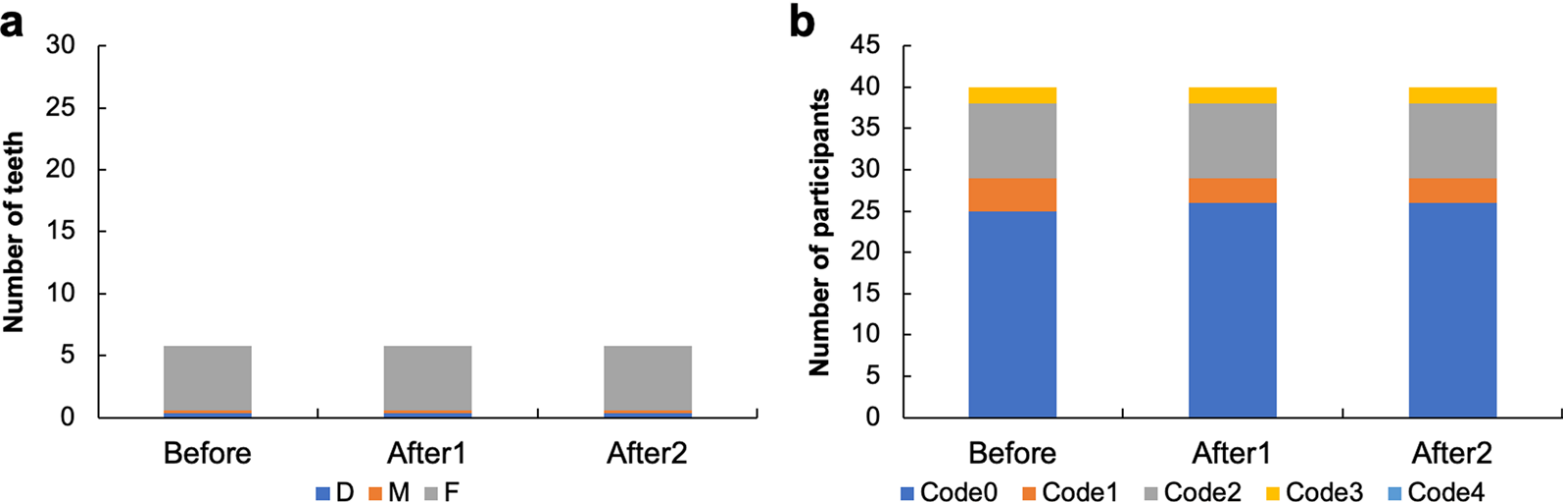
Oral examination. **a** Decayed, missing, and filled teeth (DMFT) index and **b** Community Periodontal Index (CPI). The scores adopted for DMF were as follows: carious teeth received the letter D, missing teeth received the letter M, and filled, or processed teeth that had no carious teeth received the letter F. The periodontal status of the participants was recorded using the CPI. The scores adopted for CPI evaluation were as follows: 0, healthy periodontium; 1, presence of bleeding on probing; 2, presence of calculus; 3, 4–5 mm pocket; 4, 6 mm pocket or larger

The mean DMFT values were D (mean ± SD teeth; 0.4 ± 1.0), M (0.2 ± 0.5), and F (5.2 ± 4.0). The average DMFT index before and after vaccination did not change for any participant (Fig. 2). To understand the actual condition of periodontal disease, CPI was used. A comparison of CPI before and after vaccination revealed little change in CPI and no change in the number of participants with code 4 (Fig. 2).

### Species richness and diversity (alpha diversity)

All 120 samples were sequenced using MiSeq, and 7,073,846 total sequences were amplified from the Before, After1, and After2 groups, ranging from a minimum of 14,347 to a maximum of 175,709 sequences per sample, with a mean of 52,150 sequences per sample. The sequence data generated and analyzed in this study were deposited in the DNA Data Bank of Japan (DDBJ) under the BioProject accession number PRJDB12809. The following summarizes our data: PSUD ID; PSUB 016463; and PRJDB12809.

**Fig. 3:**
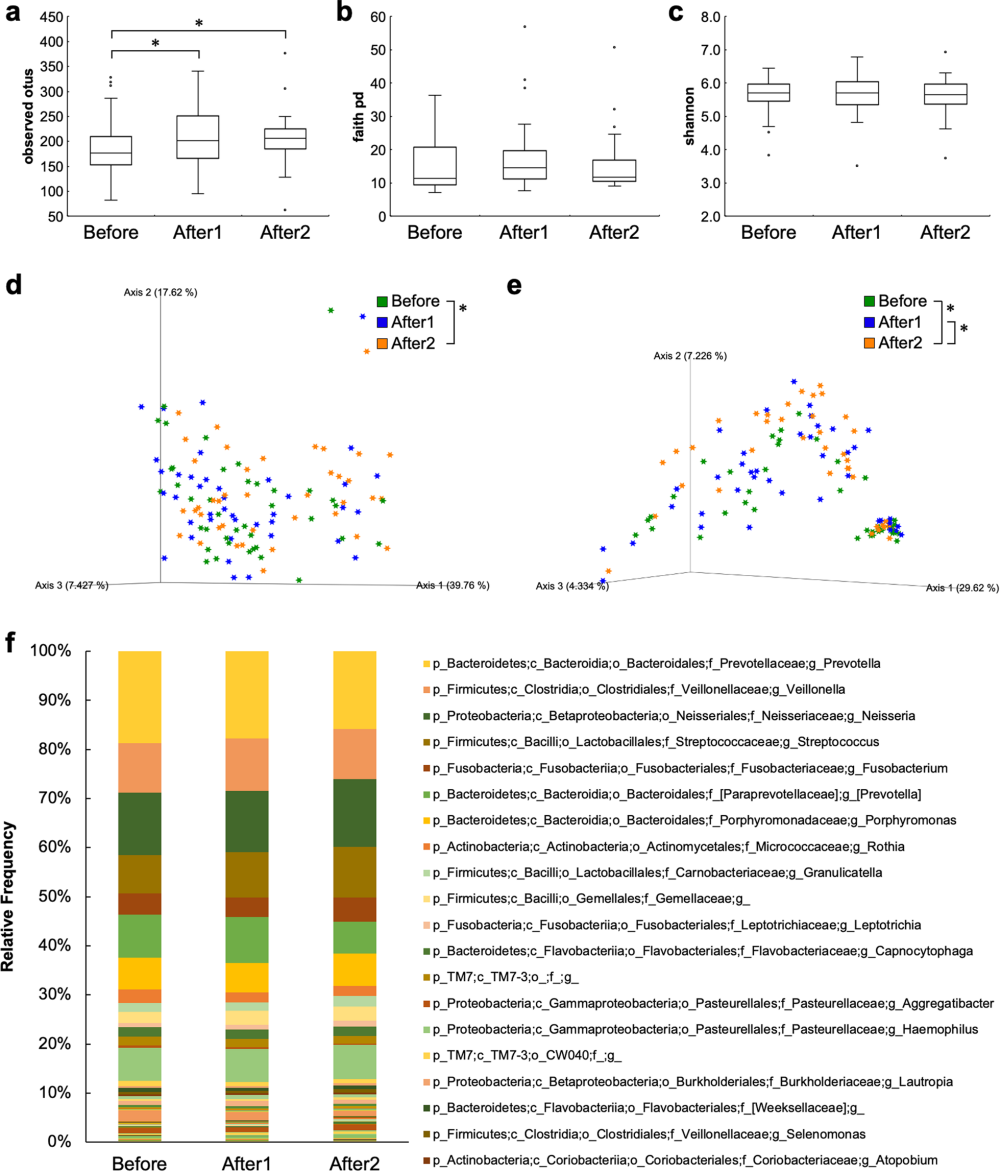
Oral microbiomes before and after COVID-19 vaccination. The diversity of oral bacterial microbiomes analyzed using **a** alpha diversity, **b** faith PD, and **c** Shannon index among the Before, After1, and After2 groups. The differences in diversity among the Before, After1, and After2 groups using principal component analysis (PCoA) of **d** weighted UniFrac distance metric and **e** unweighted UniFrac distance metric **f** QIIME2 analysis to estimate the abundance of bacterial genera. p-value less than 0.05 was considered statistically significant (*p < 0.05)

To evaluate the mean diversity of species in different sites or habitats within a local scale in each participant, alpha diversity was analyzed. The observed OTUs were significantly higher in the After1 and After2 groups than in the Before group (p = 0.040 and p = 0.007, respectively; Fig. 3a). No significant differences were observed in the Faith’s PD and the Shannon index among the Before, After1, and After2 groups (Fig. 3b, c).

### PCoA of weighted and unweighted UniFrac (beta diversity)

To evaluate the diversity differences among the Before, After1, and After2 groups, PCoA of UniFrac distance was performed. PCoA plots demonstrated clustering among the groups. The weighted UniFrac distance metric differed significantly between the Before and After2 groups based on PERMANOVA (p = 0.020; Fig. 3d). The unweighted UniFrac distance metric differed significantly between the Before and After2 groups and between the After1 and After2 groups based on PERMANOVA (p = 0.032; Fig. 3e).

### Oral bacterial taxonomy for saliva

QIIME2 detected a total of 142 different bacterial genera in the Before, After1, and After2 groups. The most abundant genus among all samples was *Prevotella*, followed by *Neisseria*, *Veillonella*, and *Streptococcus* (Fig. 3f). At the genus level, the ANCOM test revealed *Bacteroides* as a differentially abundant genus among the Before, After1, and After2 groups (W = 138); the genus was present at a lower proportion in the After1 and After2 groups than in the Before group (Table 1). Feature volatility and LME analyses were performed on the highly important bacterial genera identified by the volatility plot. Four bacterial genera were identified as being significantly differentially abundant among the Before, After1, and After2 groups (p < 0.05). Vaccination increased the abundance of the genera *Lachnoanaerobaculum*, *Moryella*, *Parvimonas*, and *Peptostreptococcus* (Table 2).

**Table 1:** The Analysis of composition of microbiomes (ANCOM) results and percentile abundances of features in each group

	Median percentile abundance			Max percentile abundance			W
	Before	After1	After2	Before	After1	After2	
p_Bacteroidetes;c_Bacteroidia;o_Bacteroidales;f_Bacteroidaceae;g_Bacteroides	6.0	1.0	1.0	29.0	4.0	6.0	138

At the genus level, the ANCOM test revealed *Bacteroides* as a different genus among Before, After1, and After2 groups; the genus was present in a lower proportion in the After1, and After2 groups than in the before group

**Table 2:** Linear mixed-effects model results for bacterial taxa among the Before, After1, and After2 groups

Bacterial taxa	Estimate	SE	Z-score	P-value
k_Bacteria;p_Firmicutes;c_Clostridia;o_Clostridiales;f_Lachnospiraceae;g_Lachnoanaerobaculum	0.000	0.000	2.082	0.037
k_Bacteria;p_Firmicutes;c_Clostridia;o_Clostridiales;f_Lachnospiraceae;g_Moryella	0.000	0.000	2.401	0.016
k_Bacteria;p_Firmicutes;c_Clostridia;o_Clostridiales;f_[Tissierellaceae];g_Parvimonas	0.000	0.000	2.577	0.010
k_Bacteria;p_Firmicutes;c_Clostridia;o_Clostridiales;f_Peptostreptococcaceae;g_Peptostreptococcus	0.000	0.000	2.292	0.022

### Alteration of microbial function

**Fig. 4:**
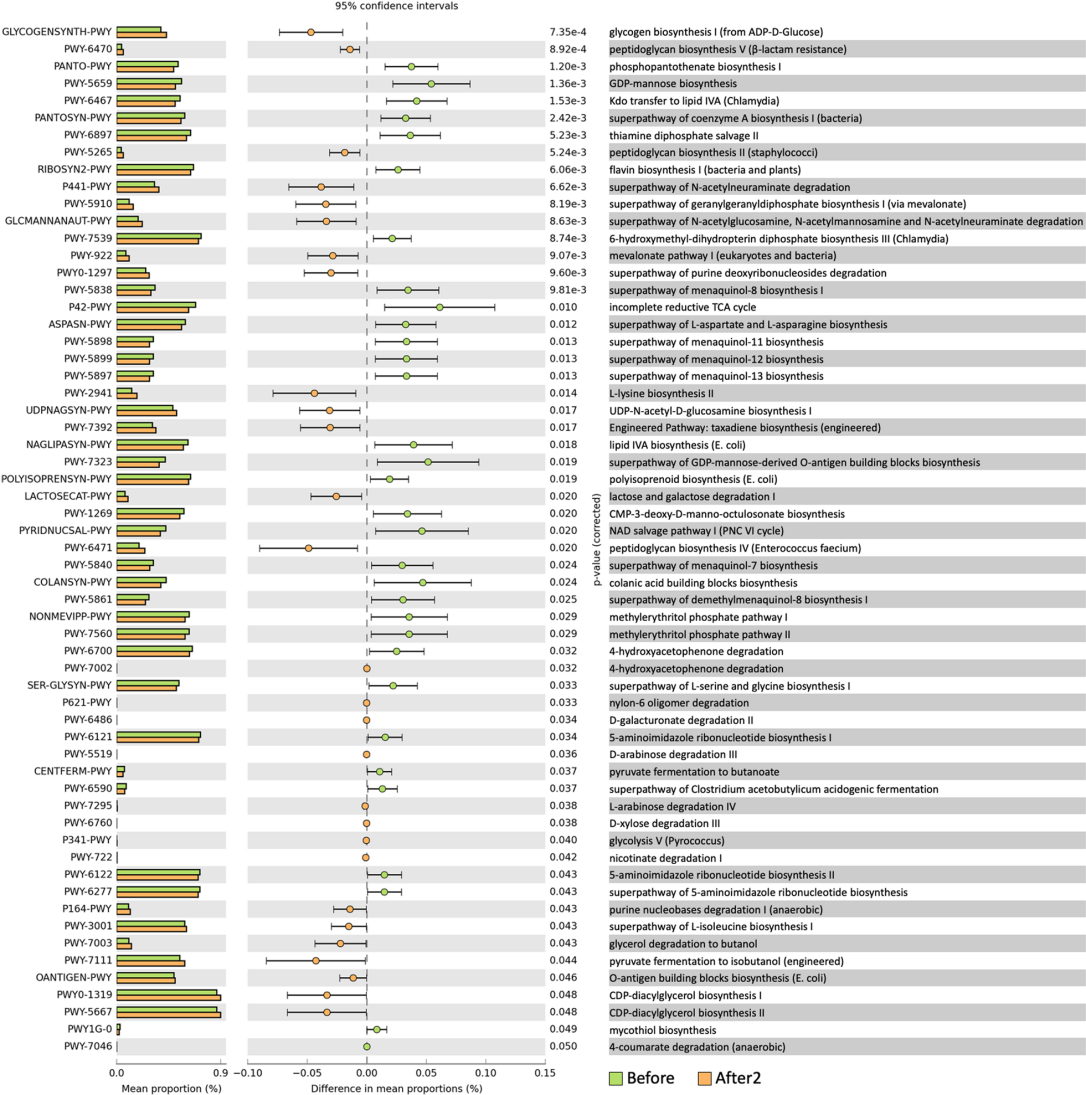
Alteration in MetaCyc pathways. Sixty MetaCyc pathways were significantly altered in the After2 group compared with those in the Before group; 28 MetaCyc pathways increased, whereas 32 decreased following COVID-19 vaccination

In all, 60 MetaCyc pathways were significantly altered in the After2 group compared with those in the Before group. Twenty-eight MetaCyc pathways, including GLYCOGENSYNTH-PWY and PWY-6470, were increased, whereas 32 pathways, including PANTO-PWY and PWY-5659, were decreased with COVID-19 vaccination (Fig. 4). Additionally, the analysis identified the highest number of pathways in the After2 samples compared with those obtained in the Before samples.

## Discussion

In this study, we performed comprehensive analyses of the saliva microbiome using next-generation sequencing (NGS) before and after COVID-19 vaccination. In addition, we examined the DMFT index and CPI. We found a significant difference in the alpha diversity (observed OTUs) and beta diversity of the flora among the Before, After1, and After2 groups. Consistent with our study, several other studies have demonstrated the alterations in the diversity of the oral and gut microbiomes through comprehensive analyses using NGS [11, 12]. For example, increased diversity of the oral microbiota has been found in patients with periodontal diseases and dental caries [13], whereas decreased diversity has been shown in patients with smoking habits and oral cancer [14, 15]. Decreased gut microbiota diversity has been found in pathological and non-beneficial conditions, including host aging, dietary changes, and psychological stress [12]. Obesity, high-energy feeding, and aging are most consistently associated with decreased gut microbial diversity [16, 17]. Moreover, patients with obesity show a significant reduction in oral and gut microbiota diversity [18]. Oral microbiota diversity is mainly affected by local factors such as the status of periodontal disease and dental caries. In this study, saliva samples were collected from dental professionals who properly managed their own oral conditions after periodontal and dental caries status was examined. The analyses revealed no significant increases in the proportion of cariogenic bacteria, such as *Streptococcus* spp. and periodontal bacteria, such as *Porphyromonas* spp. The clinical indices, including the DMFT index and CPI, did not significantly differ during the experimental period. These results suggest that COVID-19 vaccination does not affect the onset or progression of oral pathogenic bacteria, dental caries, or periodontal disease.

The local factors affecting the diversity of the oral microbiota were minimal in our study. Therefore, the increased diversity of the oral microbiota in our study might not be greatly affected by local factors. A previous study showed that oral microbiota diversity was markedly lower in the modern population than in the historical population, indicating that low diversity might contribute to chronic oral disease with a post-industrial lifestyle [19]. Therefore, it can be inferred that the increased diversity of the oral microbiota following vaccination could benefit oral health.

ANCOM showed that the abundance of the genus *Bacteroides* was significantly lower after vaccination than before vaccination. *Bacteroides* spp. are normal microbiota of the oral cavity and the gut [20]. Although the members of the genus *Bacteroides* are not pathogenic with respect to particular oral diseases, they can cause opportunistic infections. Several studies have shown the relationship between its abundance and COVID-19 severity. SARS-CoV-2 uses the angiotensin-converting enzyme 2 (ACE2) receptor, highly expressed in various body organs, including oral and nasal mucosa, the lung, heart, and gastrointestinal tract, to enter the host [21, 22]. In mice, ACE2 expression in the intestine is suppressed by *Bacteroides* spp. [23]. Patients with COVID-19 have an altered fecal microbiome, and the fecal *Bacteroides* spp. content is inversely correlated with COVID-19 severity [24]. These studies indicated that if oral *Bacteroides* spp. also suppress ACE2 expression, the reduction in *Bacteroides* spp. in the oral microbiome after COVID-19 vaccination might be a risk factor for increased ACE2 expression. In contrast, an abundance of the genus *Bacteroides* is also associated with a lack of endotoxin tolerance and increased autoimmune activity [25], suggesting that decreased abundance of the genus *Bacteroides* might ameliorate the inflammatory responses. Therefore, further studies are necessary to clarify the effect of the abundance of the genus *Bacteroides* in the oral microbiome on COVID-19 susceptibility.

We performed feature volatility and LME analyses to determine whether the relative abundances were impacted by the COVID-19 vaccination. Although we found four genera, including *Lachnoanaerobaculum, Moryella, Parvimonas, and Peptostreptococcus*, to be differentially abundant among the groups, the estimated values for these genera were small (0.000). These genera, belonging to the phylum Firmicutes, may be involved in glycometabolism in oral lesions, although the involvement may not be close.

We identified alterations in the abundance of 60 pathways, including the enrichment of several carbohydrate metabolism-related pathways, between the Before and After2 groups. A recent study showed high abundances of pathways related to carbohydrate metabolism in a metagenomic analysis of stool samples collected from COVID-19 vaccinees, including CoronaVac and BNT162b2 vaccinees [26]. Most of these pathways were positively correlated with the abundance of *Bifidobacterium adolescentis*. The enrichment of carbohydrate metabolism-related pathways may be a characteristic phenomenon in the vaccinees. The enrichments may be correlated with the abundance of several bacterial genera in the saliva of vaccinees. Further investigations are needed to clarify this speculation.

Understanding how SARS-CoV-2 vaccines affect the microbiome composition might provide important information about the changes in the oral environment following an immune response to SARS-CoV-2 infection. This information might allow us to establish new strategies for oral health management to combat COVID-19. However, this study analyzed the samples at a single time point before the vaccination, and the exact composition of the oral microbiome might vary in different time points. Therefore, further investigations including samples collected at different time points before the vaccination could provide more reliable information on temporal fluctuations not attributable to COVID-19, reveal the influences of COVID-19 vaccination on oral and gut microbiome compositions, and clarify the underlying mechanisms.

## Conclusions

The present study demonstrated alterations in the oral microbiome after COVID-19 vaccination using NGS. It showed a significant increase in oral bacterial diversity and a significant decrease in the proportion of the genus *Bacteroides* after COVID-19 vaccination.

## Data Availability

The datasets analyzed during the current study are available from the corresponding author on reasonable request.

## Abbreviations

ANCOM: Analysis of composition of microbiomes
CPI: Community Periodontal Index
DMFT: Decayed, missing and filled tooth
NGS: Next-generation sequencing
OTU: Operational taxonomic unit
PCoA: Principal coordinate analysis
PERMANOVA: Permutational multivariate analysis of variance
QIIME2: Quantitative Insights into Microbial Ecology 2
PICRUSt2: Phylogenetic investigation of communities by reconstruction of unobserved states
STAMP: Statistical analysis of metagenomic profiles
